# The RNA Binding Protein Igf2bp1 Is Required for Zebrafish RGC Axon Outgrowth *In Vivo*

**DOI:** 10.1371/journal.pone.0134751

**Published:** 2015-09-01

**Authors:** John A. Gaynes, Hideo Otsuna, Douglas S. Campbell, John P. Manfredi, Edward M. Levine, Chi-Bin Chien

**Affiliations:** 1 Program in Neuroscience, University of Utah Medical Center, Salt Lake City, Utah, United States of America; 2 Department of Neurobiology and Anatomy, University of Utah Medical Center, Salt Lake City, Utah, United States of America; 3 Department of Ophthalmology/Visual Sciences, John A. Moran Center, University of Utah Medical Center, Salt Lake City, Utah, United States of America; 4 Max Planck Institute for Brain Research, Frankfurt am Main, Germany; 5 Sfida BioLogic, Inc., Salt Lake City, Utah, United States of America; NIH/NEI, UNITED STATES

## Abstract

Attractive growth cone turning requires Igf2bp1-dependent local translation of β-actin mRNA in response to external cues *in vitro*. While *in vivo* studies have shown that Igf2bp1 is required for cell migration and axon terminal branching, a requirement for Igf2bp1 function during axon outgrowth has not been demonstrated. Using a timelapse assay in the zebrafish retinotectal system, we demonstrate that the β-actin 3’UTR is sufficient to target local translation of the photoconvertible fluorescent protein Kaede in growth cones of pathfinding retinal ganglion cells (RGCs) *in vivo*. Igf2bp1 knockdown reduced RGC axonal outgrowth and tectal coverage and retinal cell survival. RGC-specific expression of a phosphomimetic Igf2bp1 reduced the density of axonal projections in the optic tract while sparing RGCs, demonstrating for the first time that Igf2bp1 is required during axon outgrowth *in vivo*. Therefore, regulation of local translation mediated by Igf2bp proteins may be required at all stages of axon development.

## Introduction

Axon development involves several distinct processes that depend on the influence of external cues during outgrowth (which includes axon formation [1–3] and extension [4]), pathfinding [5], and synapse formation [6]. During pathfinding, outgrowth is directed by the growth cone, which navigates by turning in response to external guidance cues [5]. Growth cone receptor signaling triggers asymmetric changes in actin dynamics within the growth cone that drive extension of filopodia in the direction of attractive cues [5,7]. Local translation of mRNAs in the growth cone facilitates a rapid turning response that is autonomous from the cell body [8–11].

Attractive growth cone turning requires local translation of β-actin, and insulin-like growth factor 2 mRNA-binding protein 1 (Igf2bp1) is a candidate mediator of this process. Igf2bp1 is a member of the VICKZ family of mRNA binding proteins, and is also commonly known as zipcode-binding protein 1 (ZBP1), Vg1 mRNA-binding protein 1 (Vg1RBP/Vera), and human Imp1. *In vitro* studies have shown that netrin-1 [12,13], neurotrophin-3 (NT-3) [14,15], nerve growth factor (NGF) [16] and brain-derived neurotrophic factor (BDNF) [13,17] promote Igf2bp1-dependent localization and translation of β-actin mRNA in growth cones. Within the β-actin mRNA, the 3’UTR is sufficient to target Igf2bp1-dependent local translation of reporter mRNA in *Xenopus laevis* retinal ganglion cells (RGCs) [12,18], as well as mouse and rat cortical neurons [13]. In the cell soma, the third and fourth KH domains of Igf2bp1, which together constitute the KH34 domain, bind directly to a two-part sequence [19,20], designated the zipcode, in the β-actin 3’UTR [19–24]. Igf2bp1 represses translation during anterograde transport to the growth cone in a ribonucleoprotein (RNP) complex [12–15,25,26]. Translation of β-actin is activated when Src tyrosine kinase phosphorylates the Y396 residue in Igf2bp1 [4,13,17]. Either loss of Igf2bp1 [13] or disruption of its interaction with the β-actin 3’UTR [12,17] prevents localization and translation of β-actin mRNA in growth cones, and inhibits attractive turning. In addition, Igf2bp1 function is required for migration of chick embryonic fibroblasts [19,24,27], and tumor cells [28].

The importance of Igf2bp1 function *in vivo* has not been studied extensively. Knockdown of the Igf2bp1 ortholog Vg1RBP in *Xenopus laevis* embryos caused neural tube defects and impaired migration of neural crest cells [22,29]. Igf2bp1^-/-^ mice rarely survive past birth and although organ development is impaired [30], axon tracts in these embryos have not been analyzed. While axon tracts appear normal in Igf2bp1^+/-^ mice, filopodia are shorter and regeneration of injured axons is impaired [26]. A recent study in *Xenopus laevis* found that knockdown of Vg1RBP with an anti-sense morpholino oligonucleotide (MO) decreased retinal ganglion cell (RGC) axon terminal branching on the optic tectum *in vivo*; however, long-range navigation of RGC axons to the tectum was not affected [31]. Therefore, a requirement for Igf2bp1 function during axon outgrowth or pathfinding has not been established *in vivo*.

Here, we investigate the importance of the β-actin 3’UTR and Igf2bp1 function *in vivo*. We use the zebrafish retinotectal system, which is formed by RGC axons that exit the eye ventrally, cross the midline and then project dorsally through the optic tract to the contralateral tectum [32] to show for the first time that the β-actin 3’UTR is sufficient to target local translation of Kaede in pathfinding RGC growth cones. Impairment of Igf2bp1 caused a decrease in the number of central RGCs and increased retinal cell death. Mosaic expression of a phosphomimetic form of Igf2bp1 (Igf2bp1^Y399E^) in RGCs inhibited the growth of RGC axons, showing for the first time that Igf2bp1 function is required for axon outgrowth *in vivo*.

## Results

### The zebrafish β-actin 3’UTR is sufficient to target local translation of Kaede in RGC growth cones *in vivo*

**Fig 1:**
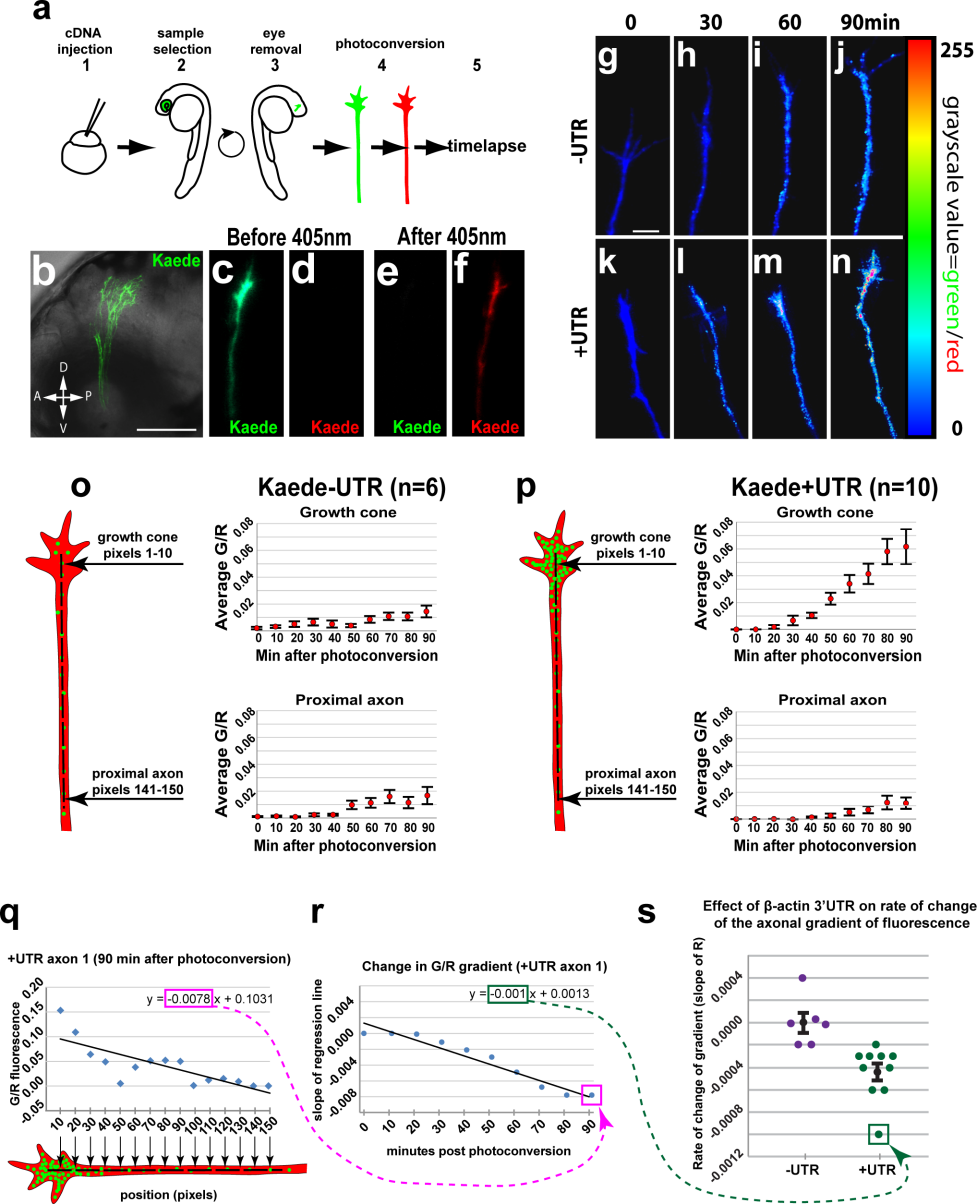
The β-actin 3’UTR is sufficient for local translation of Kaede in RGC growth cones *in vivo*. (a) Steps for *in vivo* timelapse assay: 1- cDNA injections, 2- selection of embryos with strong Kaede expression in the eye at 2dpf, 3- dissections to remove right eye and drain yolk, 4- photoconversion of Kaede in RGC axons, 5- timelapse. (b) Confocal projection (40x water lens) of a live 3 dpf embryo with Kaede expression in RGC axons in the optic tract (green). (c-f) Confocal projections of the green and red channels of one axon before (c, d) and after photoconversion (e, f). (g-n) Confocal projection from timelapse of one–UTR axon (g-j) and one +UTR axon (k-n) with green to red fluorescence ratio represented by color map. (o, p) Average green to red fluorescence intensity ratio throughout timelapse in growth cones (pixels 1–10) and proximal regions (pixels 141–150) of–UTR (n = 6) and +UTR (n = 10) axons. A one-way ANOVA (p = 0.0012) with a Tukey HSD test (p<0.05) 90 min after photoconversion showed significantly higher green to red ratio in +UTR growth cones compared to–UTR growth cones at 90 min after photoconversion. (q) The green-to-red ratio in a representative +UTR axon (axon 1) plotted against the distance from the growth cone at a representative time (90 min) after photoconversion. The slope of a linear regression to these data (outlined with a magenta rectangle) reflects the spatial gradient of green-to-red ratio 90 minutes after photoconversion. (r) Change in spatial gradient of green-to-red ratio in representative axon 1 throughout the timelapse. The point labeled Q is the slope of the linear regression in panel q, which was determined at 90 min. The other points in this graph were similarly determined in axon 1 at 10 minute intervals throughout the timelapse assay. The slope of a linear regression to these data (outlined with a green rectangle) reflects the rate of change of the gradient of green-to-red fluorescence along axon 1. (s) Rates of change in gradients of green-to-red fluorescence in all assayed axons. The point labeled R is the slope of the linear regression in panel r, which was determined in axon 1. The rates of change of the axonal gradient for the −UTR (n = 6) and +UTR (n = 10) axons were significantly different (Mann-Whitney U test, p = 0.0002). Scale bars are 100 μm (b) and 5 μm (g).

The β-actin 3’UTR was previously shown to be sufficient for netrin-1 induced local translation of the photoconvertible fluorescent protein Kaede in RGC growth cones in *Xenopus laevis* retinal explants [12,18]. In this assay, exposure of 405 nm light stably converts the Kaede protein’s fluorescence emission from 518 nm (green) to 582 nm (red). The rate of emergence of unconverted Kaede protein, arising by *de novo* translation can then be measured. A similar assay was used in cultured neurons from Igf2bp1^-/-^ mice to show that netrin-induced local translation requires the mutual functions of the β-actin 3’UTR and Igf2bp1 [13]. Since the zebrafish retinotectal system offers unique *in vivo* imaging capabilities [33–36], we adapted this assay to investigate the importance of Igf2bp1-dependent local translation of β-actin during RGC axon pathfinding in the optic tracts of live Tg(*isl2b*:Kaede-β-actin3’UTR) transient transgenic embryos (Fig 1A–1L). The *isl2b* promoter drives robust transgene expression in RGCs [37], and the β-actin 3’UTR was attached directly downstream from the Kaede stop codon (+UTR). Importantly, we developed a quantification method that compared changes in fluorescence in growth cones to changes along the axon rather than simply measuring green fluorescence inside the growth cone (see methods). Therefore, we can confidently determine whether an increase in native green Kaede in the growth cone arises from local translation.

**Fig 2:**
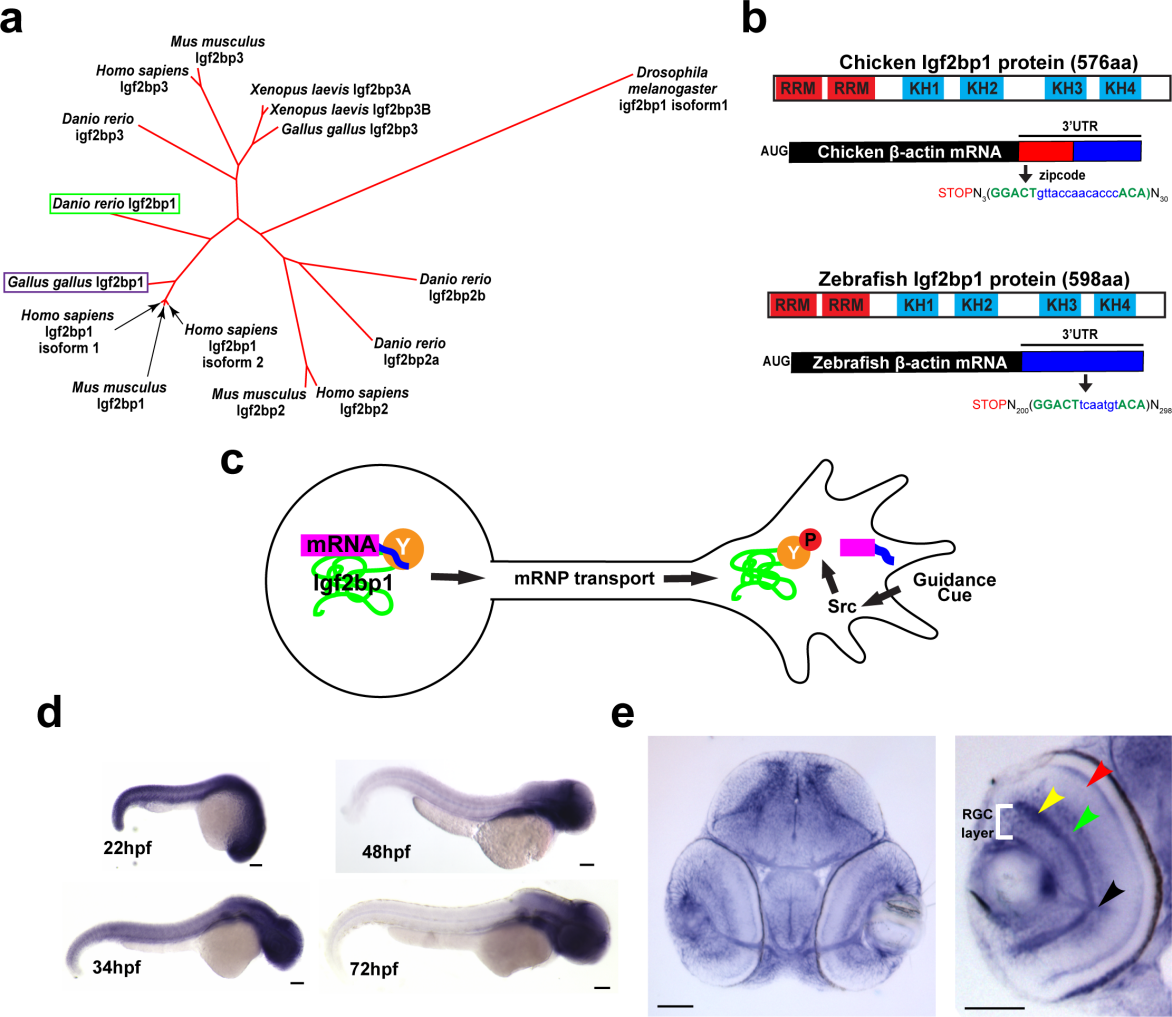
Igf2bp1, the zebrafish ZBP1 ortholog, is expressed in RGCs during axon pathfinding. (a) Phylogenetic tree generated with ClustalW, showing that of the four Igf2bp genes in *Danio rerio* Igf2bp1 is the most closely related to *Gallus gallus* Igf2bp1 of the four Igf2bp genes in *Danio rerio*, based on amino acid sequence. b) Schematic of zebrafish and chicken Igf2bp1 and β-actin mRNA, illustrating the conservation of protein domains and the presence of a bipartite nucleotide sequence in *Danio rerio* mRNA that is homologous to the zipcode sequence of chicken. These similarities at both the protein and mRNA levels suggest that in zebrafish, as has been shown in chicken, KH34 binds the putative zipcode sequence in the β-actin 3’UTR. (c) Schematic of mechanism for localization of β-actin mRNA by Igf2bp1 predicted to be conserved in zebrafish RGCs *in vivo*. (d) Wholemount *in situ* hybridizations performed on 22 hpf, 48 hpf, 34 hpf, and 72 hpf embryos show the location of Igf2bp1 mRNA expression. (e) 15 μm coronal plastic sections show Igf2bp1 mRNA expression in the RGC layer (white bracket in right panel), as well as the inner plexiform layer (yellow arrowhead), inner nuclear layer (green arrowhead), photoreceptor layer (red arrowhead) and RGC axons in the optic nerve (black arrowhead) in 3 dpf embryos. Scale bars are 100 μm (d) and 50 μm (e).

**Fig 3:**
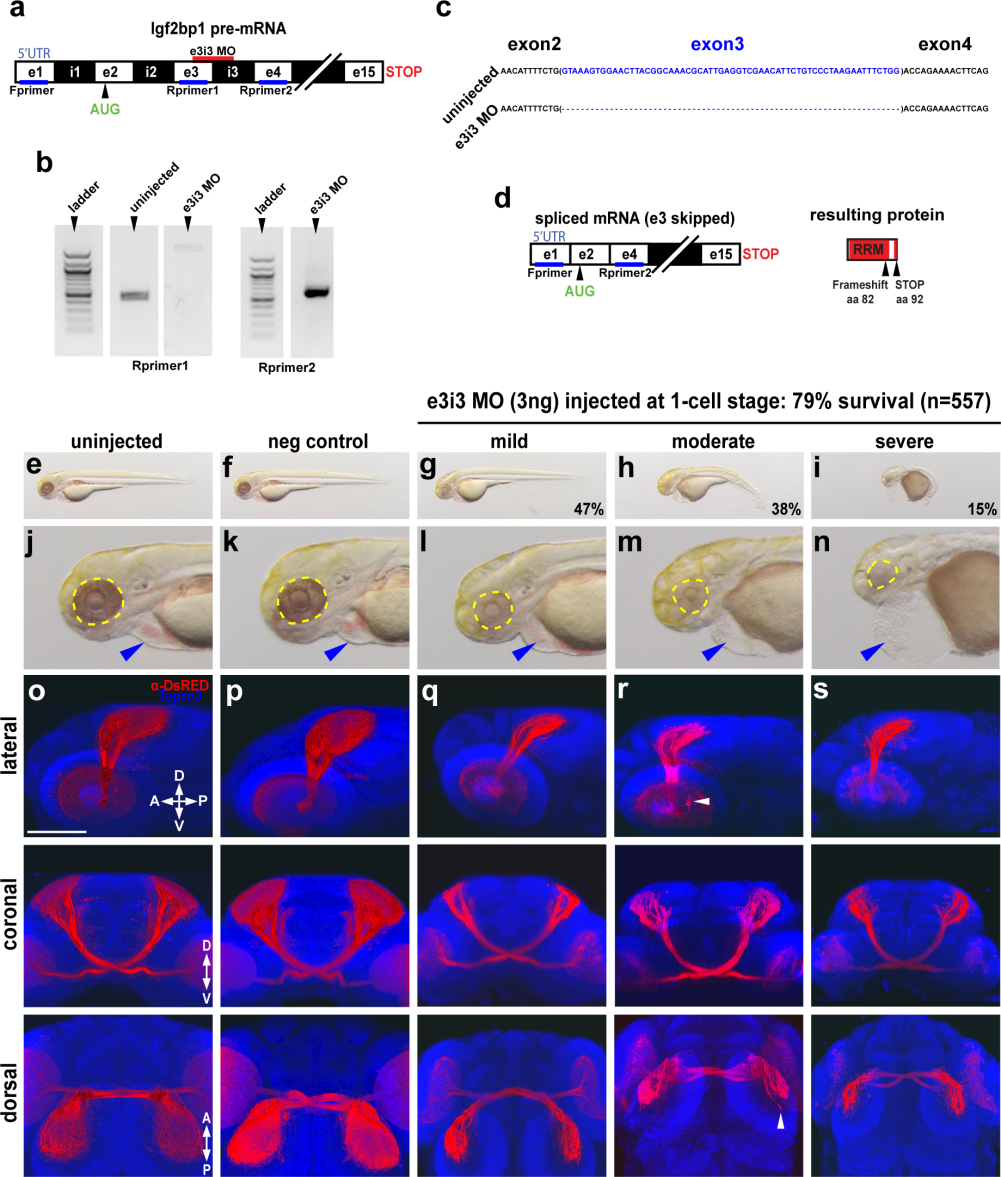
Impairment of Igf2bp1 perturbs retinotectal projections. (a) The e3i3 MO targeted to the e3i3 splice junction in Igf2bp1 pre-mRNA. (b) Left gel: forward primer targeted to exon 1 in the 5’UTR (Fprimer) and a reverse primer targeted to exon 3 (Rprimer1) yielded no detectable product as expected with exon 3 deletion. Right gel: Fprimer and a reverse primer targeted to exon 4 (Rprimer2) yielded a PCR product that was TOPO-TA cloned and sequenced (c) to verify exon 3 deletion (blank lanes were cropped out of gel image), which results in a severely truncated protein (d). (e-n) Transmitted light images of whole 3 dpf control (e,f) and e3i3 MO-injected morphants (g-i) and head regions (j-n) with eyes (yellow outline) and hearts (blue arrowheads). (o-s) 3D projections made from confocal z-stacks taken with a 30x silicone immersion lens on a confocal microscope, of Tg(*isl2b*:mCherryCAAX)^zc23^ 3 dpf embryos stained with α-DsRed (red) and counterstained with TO-PRO-3 (blue), with one example each for uninjected (o), or injected with negative control MO (p), e3i3 MO (mild (q), moderate (r), severe (s)), reconstructed from confocal z-stacks with FluoRender software, rotated to give lateral (top panels), coronal (middle panels) and dorsal (bottom panels) views. Scale bar is 100 μm.

**Fig 4:**
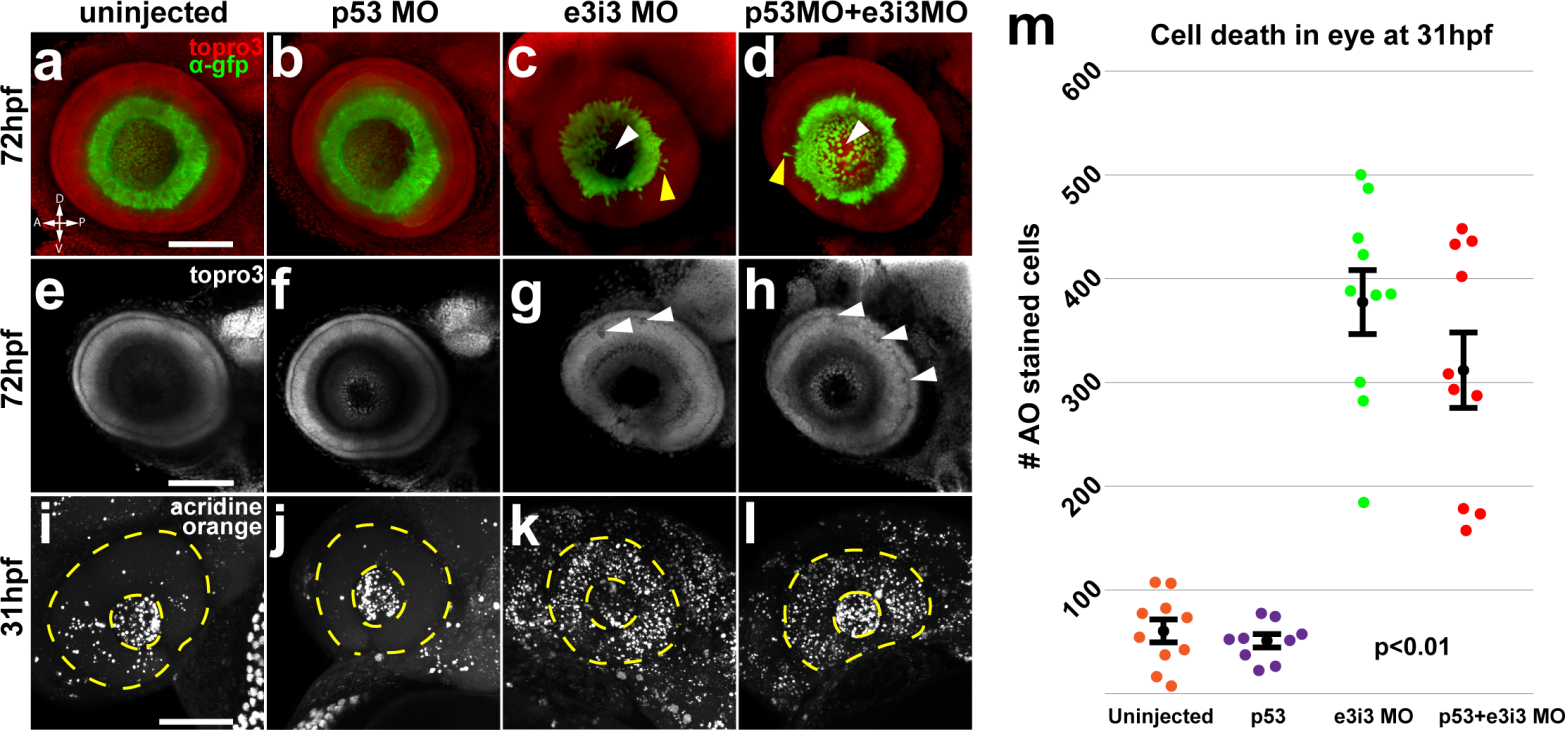
Impairment of Igf2bp1 increases cell death and layering defects in the retina. Tg(*isl2b*:GFP)^zc7^ stable transgenic embryos (a-h) were uninjected (a, e) injected with p53 MO (b, f), e3i3 (c, g), or co-injected with p53 MO+e3i3 MO (d, h). Embryos were fixed and stained with α-EGFP (green) and TO-PRO-3 (red in a-d, gray in e-h). Images are maximum intensity projections (a-d, i-l) or singles slices (e-h) of lateral views of eyes, with lens removed, taken with a confocal microscope (20x lens). Morphant eyes were missing RGCs in central retina (white arrowheads in c, d) and had displaced RGCs (yellow arrowheads in c, d). (g, h) Single z-slices have holes (white arrowheads) and abnormal layers (yellow arrowheads). (i, l) Maximum intensity projections, lateral view of AO staining in 31 hpf embryos, within the lens (inner dotted yellow circle) and retina (outer dotted yellow circle). (m) The AO-positive cells were counted in retinas alone (not including lens and extraocular tissues) from; uninjected (n = 10), p53 MO injected (n = 9), e3i3 MO injected (n = 10), and coinjected with p53 MO and e3i3 MO (n = 10) embryos. A one-way ANOVA (p<0.0001) with Tukey HSD test (p<0.01) showed that e3i3 MO injected embryos and embryos coinjected with e3i3 MO and p53 MO were both significantly different than controls, but not significantly different from each other. The black points on the graph represent mean +/- SEM. Scale bars are 50 μm.

**Fig 5:**
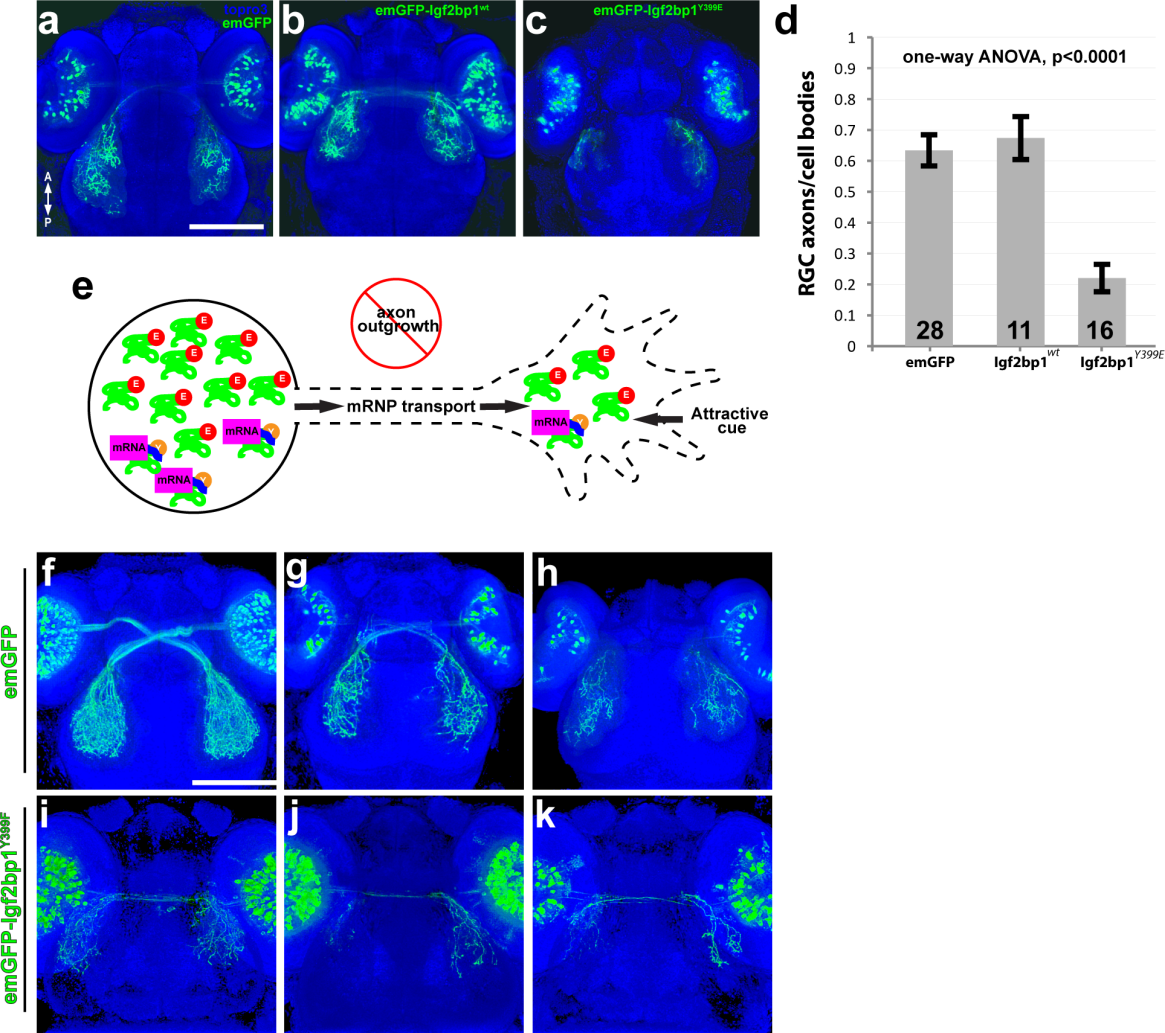
Expression of emGFP-Igf2bp1^Y399E^ in RGCs disrupts axon outgrowth *in vivo*. (a-c) Dorsal confocal projections (30x silicone immersion lens) of Tg(*isl2b*:emGFP) (a), Tg(*isl2b*:emGFP-Igf2bp1)^wt^ (b), and Tg(*isl2b*:emGFP-Igf2bp1)^Y399E^ (c) 3 dpf transient transgenic embryos. (d) Quantification of the ratio of emGFP (green) positive RGCs in the retina to emGFP positive axons on the contralateral tectum per tectum-eye pair (average +/- SEM). A one-way ANOVA (p<0.0001) with a Tukey HSD test (p<0.01) showed that the ratios were significantly different in emGFP-Igf2bp1^Y399E^ (N = 16 tectum-eye pairs, 9 animals) compared to Igf2bp1^wt^ (N = 11 tectum-eye pairs in 7 animals) and emGFP (N = 26 tectum-eye pairs, 16 animals) controls, and controls were not significantly different. (e) Schematic of the predicted mechanism for Igf2bp1^Y399E^ expression preventing axon outgrowth by interfering with endogenous Igf2bp1 by competing for mRNA transport to the growth cone without β-actin mRNA cargo, therefore decreasing the amount of β-actin mRNA available for local translation in the growth cone in response to attractive guidance cues. (f-k) Dorsal confocal projections of Tg(*isl2b*:emGFP (f-h) and Tg(*isl2b*:emGFP-Igf2bp1^Y399F^) (i-k) 3 dpf embryos. Scale bars are 100 μm.

Following cDNA injection (Fig 1A–1L) we monitored changes in the ratio of green to red fluorescence over time within single RGC axons as they grew through the optic tract. At 2 days post fertilization (dpf), embryos with mosaic Kaede expression (Fig 1A-2) were dissected to remove the right eye and drain the yolk (Fig 1A-3) and then mounted laterally at 3 dpf (Fig 1B). Axons from the left eye that grew to the dorsal optic tract near the tectum were photoconverted with a 405 nm laser (Fig 1A-4, 1C–1F) and imaged, with z-stacks acquired every 10 minutes for 90 minutes total (Fig 1A-5, 1D; S1 Movie). To analyze the data for each axon, we made a 1 pixel wide retrograde trace (150 pixels = 32 μm) starting in the growth cone (Fig 1O and 1P; S1A Fig), and then measured the ratio of green to red fluorescence in each pixel (Fig 1G–1N; S1B Fig, S2 Movie). After 90 minutes, the green-to-red ratio was significantly greater in +UTR growth cones than in both +UTR proximal axons and–UTR growth cones (Fig 1O and 1P). The green-to-red ratios in −UTR growth cones, −UTR proximal axons and +UTR proximal axons were not significantly different from one another (Fig 1O and 1P), demonstrating that the β-actin 3’UTR caused an increase in unconverted Kaede specifically in growth cones. We also quantified the temporal change of the green to red fluorescence gradient along the full length of the measurement interval in each axon (see methods), and found a significant difference between +UTR axons and −UTR axons (Fig 1Q–1S). From this we conclude that the rate of change in green to red fluorescence was significantly more asymmetric in +UTR axons compared to–UTR axons and that unconverted Kaede was not being trafficked from a distant source such as the cell soma. Therefore, the β-actin 3’UTR is sufficient for local translation of heterologous mRNA in growth cones of pathfinding RGCs *in vivo*.

### Zebrafish Igf2bp1 has a conserved structure capable of regulating local translation of β-actin mRNA

Since Igf2bp1 function is required for β-actin 3’UTR-dependent mRNA localization and translation in RGC growth cones *in vitro* [13], we predicted that Igf2bp1 functions similarly *in vivo*. A BLAST search with the amino acid sequence of *Gallus gallus* Igf2bp1 against the *Danio rerio* genome showed that the zebrafish Igf2bp1 is 81% identical to chick Igf2bp1. *Danio rerio* Igf2bp1 is a member of a four-gene family that includes Igf2bp2a, Igf2bp2b and Igf2bp3. In *Xenopus laevis*, Vg1RBP has no other family members; however, it is most similar to Igf2bp3 in *Danio rerio*. Phylogenetic analysis of all Igf2bp genes in *Danio rerio*, *Homo sapiens*, *Mus musculus*, *Xenopus laevis* and *Gallus gallus*, with *Drosophila melanogaster* Igf2bp1 as a comparison, showed that *Danio rerio* Igf2bp1 is most similar to Igf2bp1 orthologs from other species (Fig 2A).

Zebrafish Igf2bp1 has all six conserved RNA-binding domains, including RNA recognition motif (RRM) domains 1 and 2 and plextrin homology (KH) domains 1–4 of which KH34 are required for zipcode binding [13,20] (Fig 2B). We examined the zebrafish β-actin 3’UTR for the presence of a zipcode sequence and found the characteristic two part sequence, GGACT and ACA separated by 7 nucleotides, which should be able to bind KH34 [20] (Fig 2B). Therefore, we hypothesize that the zebrafish Igf2bp1 protein and β-actin mRNA are likely to interact for localization and translation through a conserved mechanism (Fig 2C).

### Igf2bp1 is expressed in RGCs during pathfinding

Wholemount *in situ* hybridization (Fig 2D) and plastic sectioning were used to assay Igf2bp1 expression (Fig 2D and 2E). Staining showed broad expression throughout the embryo at 22 hours post fertilization (hpf), and became more restricted to the head with increasing age up to 72 hpf (Fig 2D). *In situ* hybridization on 4-cell embryos showed that Igf2bp1 is maternally expressed (data not shown), similar to Igf2bp3 [38]. Coronal plastic sections of 72 hpf embryos revealed Igf2bp1 expression in the RGC layer of the retina, as well as the inner plexiform, inner nuclear and photoreceptor layers (Fig 2E). Therefore, Igf2bp1 is expressed at the right time and place to have a function in RGC axon outgrowth or pathfinding *in vivo*.

### Loss of Igf2bp1 perturbs the retinotectal projection

We hypothesized that loss of Igf2bp1 function would cause RGC pathfinding errors. 1-cell embryos were injected with a splice-blocking MO, targeted against the exon3-intron3 splice junction (e3i3) (Fig 3A). A series of injections ranging from 1–12 ng revealed that 3 ng was the maximal dose with minimal effects on organism survival at 3 dpf (data not shown). The effect of a 3 ng injection on endogenous Igf2bp1 mRNA transcript was determined by RT-PCR using RNA from injected embryos at 2 dpf, which revealed a 61 bp deletion corresponding to exon 3 (Fig 3A–3D). This deletion is predicted to cause a frame shift at amino acid 81 and an early in-frame stop codon after amino acid 91 (Fig 3D), yielding a severely truncated protein that would be unable to bind β-actin mRNA. Therefore, we conclude that e3i3 MO effectively impairs endogenous Igf2bp1 transcript.

At 3 dpf, morphants were grouped into three categories, mild, moderate and severe, based on severity of several morphological defects: decreased body size, smaller eyes, tail curvature and pericardial edema (Fig 3E–3N). In order to visualize the retinotectal projections of morphants, we injected e3i3 MO into Tg(*isl2b*:mCherryCAAX)^zc23^ embryos, which express membrane-localized mCherry in RGCs [37] (Fig 3O and 3S). Morphants were analyzed at 3 dpf when most wild-type RGC axons will have reached the optic tectum [32] and pathfinding errors are likely to be visible. Heads were imaged with a confocal microscope and 3D rendering performed using FluoRender [39] to generate lateral, coronal, and dorsal views (Fig 3O and 3S). A few morphant embryos had misrouted axons inside the retina (Fig 3R, lateral view, white arrowhead). There were also a few examples of “over-shooting” axons on the tectum (Fig 3R, dorsal view, white arrow head), similar to *Xenopus laevis* injected with Vg1RBP antisense morpholino [31]. However, the retinotectal projections of all morphant embryos consistently showed a smaller volume of neuropil from RGC axon terminal arbors on the tectum (Fig 3Q–3S) compared to controls (Fig 3O and 3P). This could be due to less elaborate branching of arbors on the tectum, as previously reported in *Xenopus laevis* [31], or it could be the result of either a defect in RGC axon outgrowth or a decreased number of RGCs in the retina. Morphants injected with another morpholino targeted to the i3e4 splice junction in Igf2bp1 pre-mRNA (i3e4 MO) gave a retinotectal phenotype similar to e3i3 MO injected morphants (S2 Fig). Therefore, we conclude that this retinotectal phenotype is specific to loss of endogenous Igf2bp1 function.

### Igf2bp1 knockdown caused a loss of central RGCs

We examined morphant retinas to determine if RGC axons were unable to exit the eye (Fig 4A–4D). In order to prevent non-specific cell death [40] we co-injected p53 MO with the e3i3 MO into Tg(*isl2b*:GFP)^zc7^ embryos, which express GFP in the majority of RGCs (Fig 4A–4D). RGC axons normally exit the eye through the optic disc in the ventral retina, where netrin is expressed [41]. When we examined confocal images of eyes in morphant embryos we did not see an accumulation of RGC axons and growth cones inside the retinas, as would be expected if RGC axons were stalled or unable to exit the eyes. However, some retinal defects were noted (Fig 4A–4H). The RGC layer was thinner and the border was less defined in morphant retinas, with several displaced GFP^+^ RGCs (Fig 4C and 4D, yellow arrowheads). Furthermore, there were large holes in the central domains of morphant retinas, where both GFP and TO-PRO-3 staining were absent (Fig 4C and 4D, white arrow heads), suggesting that RGCs were absent. Pioneer RGCs located in the central retina are the first to differentiate, and outgrowth of axons from later born RGCs is dependent on interactions with pioneer axons [37]. Therefore, loss of central RGCs could cause a reduction in the number of axons that exit the eye. There were also small holes seen in the TO-PRO-3 staining in morphant retinas (Fig 4G and 4H, white arrowheads), suggesting that cell death was occurring.

Acridine orange (AO) staining was used to visualize apoptotic cells in live embryos [42] (Fig 4I–4L). In both uninjected and p53 MO injected controls, most AO staining appeared in the lens (Fig 4I and 4J inner circles). Embryos injected with e3i3 MO alone and embryos co-injected with p53 MO and e3i3 MO all had a striking increase in AO-positive cells throughout the retina (Fig 4K and 4L outer circles). Counts of AO positive cells in retinas (S3 Movie, see methods) from confocal datasets of 31 hpf embryos using Imaris revealed a significant increase in AO-positive cells in morphant retinas compared to controls (Fig 4M). There was not a significant difference between the numbers of AO-positive cells in retinas from embryos injected with e3i3 MO alone and from embryos co-injected with e3i3 and p53 MOs (Fig 4M). Therefore, the loss of central RGCs and increased retinal cell death were likely due to specific loss of Igf2bp1 function [40].

### RGC-specific expression of dominant negative Igf2bp1 interferes with axon outgrowth *in vivo*

Phosphorylation of chick Igf2bp1 at Y396 relieves its binding to β-actin mRNA and activates translation [4,17,20,43], and the phosphomimetic Y396E mutation decreases binding to β-actin mRNA [4,20]. Based on these findings, we predicted that zebrafish Igf2bp1 with its tyrosine at 399 switched to glutamate (Igf2bp1^Y399E^) would be unable to bind or transport β-actin mRNA, but would still assemble with RNP granules, and its overexpression in RGCs would therefore interfere with endogenous Igf2bp1 function. To test this in an RGC-specific manner, we expressed Igf2bp1 or Igf2bp1^Y399E^ under the control of the *isl2b* promoter (S3C and S3D Fig) by injecting expression constructs at the one-cell stage. To visualize expression in individual RGCs, N-terminal fusions were generated with emerald GFP (emGFP control) that was engineered to minimize multimer formation. Specifically, lysine was substituted for alanine at position 207, which promotes the formation of a monomeric, fluorescent protein (see methods). To further limit the possibility of steric interference with Igf2bp1 function, we also added an inert and flexible peptide linker between emGFP and Igf2bp1 (see methods, S3A Fig). As expected, the resulting transient transgenic embryos exhibited mosaic expression, so we compared embryos with a moderate number of emGFP expressing RGCs to facilitate counting and to minimize non-autonomous effects of Igf2bp1 interference. We did not observe misrouted RGC axons at any point in the retinotectal trajectory, nor was there evidence of axonal degradation (Fig 5C). Rather, emGFP-Igf2bp1^Y399E^ expression in RGCs decreased the number of emGFP^+^ axons on the tectum relative to the number of emGFP^+^ cell bodies in the contralateral retina, compared to controls (Fig 5A–5C); that is, the ratio of tectum-projecting axons to cell bodies was significantly lower in RGCs that express the dominant negative Igf2bp1^Y399E^ (Fig 5D, S4 Fig). From this we conclude that expression of Igf2bp1^Y399E^ in RGCs prevented axon outgrowth (Fig 5E). Expression of a similar protein with a non-phosphorylatable mutation (emGFP-Igf2bp1^Y399F^) in RGCs also reduced the number of axons that grew to the tectum at 3 dpf (Fig 5F–5K). Since expression of both mutant proteins with Y399 mutations gave similar phenotypes, we conclude that loss of endogenous Igf2bp1 function interfered with RGC axon outgrowth.

## Discussion

This study utilized the zebrafish retinotectal system as a model to investigate Igf2bp1-dependent local translation of β-actin in developing RGC axons *in vivo*. We demonstrated that the zebrafish β-actin 3’UTR is sufficient to target local translation of Kaede to pathfinding RGC growth cones in the optic tract *in vivo*. This result is consistent with the results of other studies that used the β-actin 3’UTR [12,13,18] or the neural fold protocadherin (NFPC) 3’UTR [44]. However, our study is the first to demonstrate this in a *bona-fide in vivo* context. *In vitro* studies have shown an increase in local translation of Kaede [12,18] or Dendra2 [13] in growth cones only when netrin is manually applied to axons growing in culture medium. Our *in vivo* assay showed that endogenous amounts of guidance cues are sufficient to increase β-actin 3’UTR-dependent local translation of Kaede in RGC growth cones. Furthermore, RGC growth cones encounter many different attractive and repulsive cues *in vivo* [5]. However, we were able to measure an increase in local translation of Kaede in +UTR growth cones even in the presence of inhibitory signals, adding further significance to our *in vivo* result and demonstrating that β-actin 3’UTR-dependent local translation is a fundamental process that occurs during RGC axon pathfinding *in vivo*.

Since netrin-1a [45,46] and BDNF [47] are expressed in the retina and along the retinotectal projection throughout RGC axon development *in vivo*, it is possible that these guidance cues trigger Igf2bp1-dependent local translation, as suggested by *in vitro* studies [12,13,17]. Furthermore, the structure of zebrafish Igf2bp1 protein is similar to orthologs known to regulate localization and translation of β-actin mRNA in neurons [20]. In addition, the zebrafish β-actin 3’UTR contains a two-part sequence that should be able to bind KH34. Finally, Igf2bp1 is expressed in zebrafish RGCs during axon development. Therefore, it is likely that Igf2bp1-dependent local translation of β-actin is utilized in pathfinding RGC growth cones *in vivo*.

The broad expression pattern of Igf2bp1 during embryonic development suggests that Igf2bp1 function is not restricted to axon development. Igf2bp1 mRNA is expressed strongly throughout the developing zebrafish nervous system, similar to Vg1RBP expression in *Xenopus laevis* [38] and Igf2bp1 in mouse [30]. Igf2bp1 is expressed maternally (data not shown) and throughout the entire embryo at 22 hpf prior to RGC differentiation. Interestingly, cell death in e3i3 morphants was abundant and broad, similar to the Igf2bp1 mRNA expression pattern. Cell death in e3i3 morphants was not rescued by co-injection with p53 MO, suggesting that the reduced cell survival results from impaired Igf2bp1 function. Given its roles in cell migration, morphogenetic movements and binding to multiple mRNAs [19,21,22,24,29,48,49], Igf2bp1 is likely to have multiple functions in embryonic development.

Embryos injected with the e3i3 MO had atrophic retinotectal projections with thin optic nerves and diminished tectal neuropil. Although the severity of this phenotype increased with the severity of gross morphological defects, all morphant retinotectal projections that were analyzed were noticeably perturbed, even in embryos with mild gross morphological defects. The atrophic projections may be more associated with RGC loss rather than axon outgrowth because increased cell death in morphants may have decreased the number of RGCs in the retina. However, the dramatic loss of central RGCs in morphant retinas suggests that Igf2bp1 may have a specific function in central RGCs, resulting in a loss of pioneer axons and a decrease in overall axon outgrowth in morphants.

We observed RGC axon pathfinding errors in the retina and on the tectum of embryos injected with e3i3 MO. However, these were rare occurrences as the majority of axons showed normal trajectories. Therefore, RGC axons were able to navigate correctly to the contralateral tectum in morphants, consistent with the study in *Xenopus laevis*, which reported that Vg1RBP was not required for long range RGC axon navigation *in vivo* [31]. In our timelapse analysis we quantified β-actin 3’UTR-dependent local translation in growth cones of axons that were in the dorsal optic tract near the optic tectum, therefore, it is possible that there is a position-dependent requirement for Igf2bp1 function in RGC axons in the dorsal optic tract and the tectum where most axonal defects were observed, similar to NFPC [44]. While RGC-specific expression of Igf2bp1^Y399E^ caused an axon outgrowth defect, the RGC axons that did grow out from the retina navigated correctly to the contralateral tectum, further supporting the idea that Igf2bp1 is not required during pathfinding *in vivo* and that local translation of β-actin in RGCs is only required in the dorsal optic tract for termination and arbor formation on the tectum.

Expression of mutated Igf2bp1^Y399E^ and Igf2bp1^Y399F^ in RGCs revealed that Igf2bp1 function is required for axon outgrowth *in vivo*. Previous *in vitro* studies have suggested that Igf2bp1 is required for attractive growth cone turning but not axon outgrowth [12], and that local translation of β-actin promotes axon branching but not elongation [50], yet there is also *in vitro* evidence that loss of Igf2bp1 function perturbs axon outgrowth [4]. Interestingly, chicken Igf2bp1 with the non-phosphorylatable Y396F mutation is transported to axons similarly to wild-type Igf2bp1 but it decreases growth cone turning towards netrin in mouse cortical neurons and also decreases neurite outgrowth in primary hippocampal neurons and neuroblastoma cells [4,12,13]. Thus, while the phosphomimetic mutation has an opposite effect on the molecular function of the tyrosine residue compared to a non-phosphorylatable mutation, the effects of both mutations on axon outgrowth are similar. Therefore, active phosphorylation of this conserved tyrosine residue is critical for Igf2bp1 function and is required for axon outgrowth *in vivo*.

Mosaic expression of emGFP-Igf2bp1^Y399E^ provided us with the resolution to identify and quantify the RGC axon outgrowth defect *in vivo*. We were able to visualize and count emGFP^+^ cell bodies in the retina and axons on the tectum, while images of embryos with emGFP-Igf2bp1^Y399F^ in RGCs qualitatively showed a similar effect on axon outgrowth. A similar study in *Xenopus laevis* used electroporation to express a dominant negative form of Vg1RBP (Vg1RBP^**ΔKH4**^) in RGCs *in vivo* and reported that long range RGC axon navigation was not affected [31]. However, a decrease in the ratio of axons to cell bodies may not have been visible due to the high number of RGCs expressing Vg1RBP^**ΔKH4**^, and counting was not likely to have been feasible due to extensive labeling [31]. Broad labeling of RGCs by the *isl2b*:mCherryCAAX transgene would have similarly masked an effect in the morphant embryos analyzed in the present study. Another advantage of mosaic expression of the dominant negative is that it should interfere with redundant function from other Igf2bp1 family members. Furthermore, use of the *isl2b* promoter allowed us to express the dominant negative protein specifically in RGCs, allowing us to directly examine the requirement of Igf2bp1 function in RGCs without interfering with Igf2bp1 function in earlier stages of development.

The deficit of axons from Igf2bp1^Y399E^- and Igf2bp1^Y399F^-expressing RGCs suggests that Igf2bp1's role in axon outgrowth could potentially have a role at the level of initiation. Like growth cone turning and terminal branching, initiation of axon outgrowth depends on neurite extension in response to external cues. Since netrin [41,46] and BDNF [47] are expressed in the retina during RGC axon development and can trigger Igf2bp1-dependent local translation of β-actin mRNA [12,13,17], it is conceivable that these cues initiate RGC axon formation through Igf2bp1-dependent local translation of β-actin mRNA *in vivo*. It is also possible that the deficit of axons was caused by a decreased rate of extension or increased degradation. Therefore, the scope of Igf2bp1 function during axon development *in vivo* may be wider than previously expected.

## Materials and Methods

### Ethics statement

Experimental protocols were approved by the University of Utah Institutional Animal Care and Use Committee (IACUC) and followed NIH guidelines.

### Fish

All wild-type embryos were from the Tübingen or TL strains. Transgenic strains Tg(*isl2b*:GFP)^zc7^ and Tg(*isl2b*:mCherryCAAX)^zc2^ were used [37]. All fish were raised at 28.5°C.

### Timelapse assay

A previously described local translation timelapse assay was adapted to pathfinding RGC axons in the zebrafish optic tract [12,18]. At 2 dpf, transient Tg(*isl2b*:Kaede-β-actin3’UTR-pA) or Tg(*isl2b*:Kaede-pA) embryos were sorted for bright fluorescence in RGCs, anesthetized with 0.02% tricaine (Sigma-Aldrich Co. LLC, St. Louis, MO), and mounted dorsally in drops of 1.5% low-melt agarose in E2/gentamycin. The agarose was windowed to expose eyes and covered with E3+1mM PTU (Sigma-Aldrich Co. LLC, St. Louis, MO) (0.02% tricaine). A pulled glass pipette with a short taper was used to dissect the exposed eye from the embryo. The embryos were removed from the agarose and returned to E3+PTU at 28.5°C (0.02% tricaine). Approximately 50% volume of yolk was drained from embryos by squeezing it out through a small hole torn with sharpened tungsten needles. Embryos were then recovered in E3 (1mM PTU) at 28.5°C. At 3 dpf, embryos were mounted laterally in a petri-perm (Sigma) dish in drops of low-melt agarose, with the side without an eye facing down, and covered in E3 (1mM PTU, 0.02% tricaine). Embryos with isolated RGC axons growing in the optic tract were selected for timelapse, removed from the petri-perm dish and remounted in a dish with a glass coverslip bottom. Fish were pressed gently against the bottom of the dish to maximize availability of the optic tract for imaging. The dish was placed on a heated stage at 28.5°C. RGC axons with visible growth cones were selected and the whole heads photoconverted for 2 minutes with 405 nm laser irradiation at maximum intensity, until green Kaede was undetectable and red Kaede very bright. Timelapse was begun immediately taking a z-stack every 10 minutes ten times, for a total of 90 minutes. A 40x water immersion lens was used with immersol (Fischer Scientific, Waltham MA), with a 3x zoom. All file handling and image analysis was done in ImageJ (http://imagej.nih.gov).

### Post-processing and quantification of confocal timelapse imaging datasets

RGC axons in the dorsal optic tract with bright Kaede expression and that grew noticeably toward the tectum throughout the timelapse after photoconversion were selected for quantitation (+UTR (n = 10) and −UTR (n = 6)). The timelapse confocal dataset for each axon was separated into red and green channels, for each of 10 time points after photoconversion (10 red, 10 green). In ImageJ, the red stack for one timepoint was converted to a binary mask and subtracted from the corresponding green stack. SUM projections were then generated from the red stack and edited green stack, and the green SUM projection was divided by the red SUM projection giving a ratio SUM projection, with pixel gray scale values representing the ratio of green fluorescence to red fluorescence. Using the axon in the red SUM projection as a guide, a 1 pixel wide retrograde trace was made with the simple neurite tracer plugin in Fiji (S1A Fig). The X and Y coordinates for the first 150 pixels of the trace were used to identify corresponding pixels in the ratio SUM projection, and the gray scale values for these pixels were measured and recorded with a custom ImageJ macro (S1B Fig). The gray scale values for each ten consecutive pixels (1–10, 11–20, etc.) were averaged, giving 15 values for each axon at each timepoint (S1B Fig).

### Quantification of change in axonal gradient of fluorescence

We first compared the 10 pixel averages of the ratios of the green-to-red fluorescence intensities from growth cone regions (pixels 1–10) and the proximal axon regions (pixels 141–150) for +UTR axons (n = 10) and–UTR axons (n = 6) at 90 minutes after photo-conversion (Fig 1O and 1P).

In order to quantitate the rate of change in the intensity of green-to-red fluorescence based on axonal position over time using the measurements generated by the ImageJ macro (S1 Fig, S2 Movie), the 10 pixel averages of the green-to-red fluorescence ratios from one axon at one timepoint (y-axis) were plotted against position (x-axis) starting in the growth cone (pixels 1–10) and ending in the proximal axon (pixels 141–150) (Fig 1Q), giving 10 different graphs for each axon. The slopes of the linear regression lines for each of these graphs represented the gradient of green to red fluorescence along the axon at one timepoint (Fig 1Q, magenta box). In order to determine the rate of change in the gradient for each axon, one graph was made for each axon with the slopes of the linear regression lines of the graphs described above (Fig 1Q), plotted against time (Fig 1R). The slope of the linear regression line from this 2^nd^ graph represented the rate of change in the slopes from the 1^st^ graphs over time for the axon. The slopes of the 2^nd^ graphs for +UTR axons were compared to the slopes of the 2^nd^ graphs for–UTR axons with a Mann-Whitney U test (Fig 1S).

### DNA constructs

The following cDNA constructs were constructed in pDEST-Tol2-pA2 with the Tol2 kit [51]: *isl2b*-Kaede-β-actin3’UTR-pA, *isl2b*-Kaede-pA, *isl2b*-emGFP-Igf2bp1^Y399E^ –pA, *isl2b*-emGFP-Igf2bp1^Y399F^-pA, *isl2b*-emGFP-Igf2bp1-pA, *isl2b*-emGFP-pA, and *pBSII-igf2bp1* were all constructed in pDEST-Tol2pA2 with the Tol2 kit [51]. Zebrafish Igf2bp1 coding cDNA was obtained from Open Biosystems (Accession EB781185) and used to generate pBSII-igf2bp1through conventional cloning. The emerald GFP cDNA was a gift from Scott Holly (Yale University, Department of Molecular, Cellular and Developmental Biology). Fusion PCR was used to make the A207K point mutation to generate monomeric emGFP (S3 Fig).

### Generation of monomeric emerald GFP (emGFP) fusion

In order to make an N-terminal fluorescent fusion with Igf2bp1, with bright fluorescence and minimum risk of abnormal dimerization or steric interference with Igf2bp1 function, emerald GFP with the A207K mutation [52] and a flexible inert peptide linker [53] was generated (S3 Fig). Two rounds of PCR were performed with pCS2-emGFP as a template (S3A Fig): PCR 1 with Fprimer1: 5’-GCCGTGCGGATCCCCACCATGGTGTCCAAGGGCGAGGAGCTGTTCACCGGCGTGGTGCCTATCCTGG-3’, with a BamHI site and kozak consensus, and Rprimer1: 5’- GTCGCGCTTCTCGTTAGGGTCCTTGCTCAGCTTGCTCTGGGTGCTCAGGTAGTGGTTGTC, spanning A207 with a point mutation, and PCR2 with Fprimer2: 5’- GACAACCACTACCTGAGCACCCAGAGCAAGCTGAGCAAGGACCCTAACGAGAAGCGCGAC, which is the reverse complement to Rprimer1 and also contained the A207K point mutation, and Rprimer2: 5’- GTCGCCTGCGGCCGCGGGGCCAAACC-CCTCCTCTGCCGCAGCCAATCAGAGAGCCAGGAC-3’. PCR product from each reaction was gel purified and mixed together as template for fusion PCR with Fprimer1 and Rprimer2 (S3A Fig). Fusion PCR product was gel purified, double digested with BamHI and NotI, gel purified, and treated with Antarctic phosphatase at 37°C for 30 minutes, then ligated into pCS2 and verified with restriction digest and sequenced directly to verify successful incorporation of the A207K point mutation. The pME-emGFP-linker clone was generated by a BP recombination reaction with Fprimer: 5’-GGGGACAAGTTTGTACAAAAAAGCAGGCTGTGGCCGGCCTACCACCA-TGGTGTCCAAGGGCGAGGAGCTG-3’ and Rprimer: 5’- GGGGACCACTTTGT-ACAAGAAAGCTGGGTGCTGCTTCCGCTTCCGGTGCTTCCGGTTCCGTGTCCTGGCGCGCCCTTGTACAGCTCGTCCATTCCCAG, which added a flexible 12 amino acid peptide linker [53] in frame with the emGFP coding sequence and also lacked a stop codon (S3B Fig). The pME-emGFP-linker construct was used for LR reactions (S3C Fig) to generate *isl2b*:emGFP-linker-Igf2bp1wt-pA and *isl2b*:emGFP-linker-Igf2bp1^Y399E^-pA in the pDEST-Tol2-pA2 destination vector (S3D Fig) and used for 1-cell DNA injections for transient transgenesis. The cDNA expression constructs used for the *in vivo* timelapse experiment, *isl2b*:Kaede-pA and *isl2b*:Kaede-β-actin3’UTRpA, were generated similarly by LR recombination (S3C and S3D Fig).

### Morpholinos

All morpholino oligonucleotides (MOs) were designed by and purchased from Gene Tools, LLC (Philomath, Oregon). Igf2bp1 splice-blocking oligonucleotides (e3i3 MO (5’-TCTGGTCCTGTAGA-GAAAGAAATGA-3’) and i3e4 MO (5’-TCTGGT-CCTGTAGAGAAAGAAATGA-3’)), standard negative control MO (5’-CCTCTTACCTCAGTT-ACAATTTATA-3’) and zebrafish p53 MO (5’-GCGCCATTGCTTTGCAAGAATTG-3’). Lyophilized MOs were resuspended in ddH20 at 1mM stock and stored at room temperature.

### DNA and morpholino injections

All injections were performed with an ASI pressure injector delivering 1nl into the cytoplasm of 1-cell embryos. Injected embryos were raised in E2/gentamycin for 8 hours and then in E3 (1mM PTU). DNA was injected into wild type at 25 pg with 25 pg transposase RNA in RNAse free ddH2O (0.1% phenol red). MO was injected into wild type, Tg(*isl2b*:mCherryCAAX)^zc23^, or Tg(*isl2b*:GFP)^zc7^. Injected amounts of MO were: 3 ng e3i3 MO, 1.5 ng i3e4 MO, 4.5 ng p53 MO, 6 ng negative control MO.

### Reverse transcription polymerase chain reaction

Wild-type embryo’s injected with 3ng e3i3 MO were collected at 48 hpf, and total RNA preps from 20 embryos were used to make cDNA with reverse transcription. Igf2bp1 cDNA was amplified with f-primer in exon1 (5’-CGCCAAGGTTGCTACAGT-GAAGAATATTTACCAC-3’) and either r-primer in exon3 (5’-CACTGCAGGTGTGG-TGGAATCTTTCTGATC-3’) or r-primer in exon4 (5’-CACAGTTCTCAACAGTTCC-ATATTGGGCAAG-3’). PCR product using the exon4 r-primer was gel purified and Topo-cloned for direct sequencing with M13 primers.

### Immunostaining

Embryos were selected for imaging under an Olympus SZX16 fluorescent dissecting microscope at 3 dpf and then fixed in 4% paraformaldehyde for 6 h at 4°C. Embryos were washed in phosphate buffered saline containing 0.5% Triton X-100 (PBST), permeabilized with 0.1% collagenase in 2%PBST, blocked with 0.1% new born calf serum (NCST) and then incubated with primary antibody (1:700 rabbit anti-EGFP or 1:200 rabbit anti-DsRed in NCST) for 2 d. Embryos were washed with PBST and incubated with 1:1000 TO-PRO-3 (1 μM) and secondary antibody (1:200 Alexa 488 goat anti-rabbit or Cy3 goat anti-rabbit in NCST). Embryos were washed, incubated in 50% glycerol (PBS) for 2 h then 80% glycerol (ddH_2_O) overnight and stored at -20°C. Embryos were prepared for imaging in 100% glycerol under cover glass.

### *In situ* hybridization

*In situ* hybridization was performed as previously described [54], on wild type embryos using Igf2bp1 antisense probe made from pBSII-Igf2bp. Embryos were photographed in glycerol under an Olympus SZX16 dissecting scope with SPOT camera software (SPOT Imaging Solutions, Sterling Heights, Michigan).

### Plastic sectioning

After *in situ* hybridization, embryos were incubated in 1:1 Immuno-Bed (Polysciences, Inc., Warrington, Pennsylvania in MeOH for 30 minutes and then 100% Immuno-Bed overnight. Embryos were embedded in Immuno-Bed with (1:20) solution B (EMS catalog# 14260–04). Sections of 12.5μm were made using a Reichert-Jung supercut microtome with a glass knife.

### Acridine Orange staining

A 10μm diameter bolus of acridine orange (Sigma catalog# A6014) suspended in ddH_2_O was injected into the yolk of embryos as previously described [42] 2 h before imaging. Fluorescent pictures were taken using the GFP excitation and emission filters on an Olympus SZX16 fluorescent dissecting microscope. The eyes of 31 hpf AO- stained embryos were imaged with an Olympus FV1000 confocal microscope (20x lens).

### Equipment and settings

All confocal imaging was performed with an Olympus FV1000 confocal microscope and FV10-ASW 3.1 software. Lenses used were UAPON 40x water immersion 340 (Fig 1B–1N, S1 Fig, S1 Movie and S2 Movie), UPLSAPO 30x silicone oil immersion (Figs 3J–3N, 4A–4H and 5A–5C) and UPLSAPO 20x (Fig 4I–4L, S3 Movie). Settings used for acquisition of images are listed in S1 Table. Confocal z-stacks were edited in ImageJ. 3D rendering was performed with Fluorender [39]. Gamma was applied to confocal projections in FluoRender to enhance visibility of RGC axons in Figs 1B, 3J–3N, 4A–4D and 5A–5C. Images (Fig 1G–1N) and frames (S1 Movie) are maximum intensity projections generated in FluoRender based on original gray scale values of confocal z stacks, without non-linear operations applied, such as gamma. Images in Fig 1C–1F are maximum intensity projections generated in ImageJ from confocal z stacks. Images in Fig 4E–4H are single confocal slices. Images in Fig 4I–4L are maximum intensity projections generated in ImageJ. All Quantitation’s were performed on images that reflected original gray scale values, without application of non-linear operations, such as gamma.

### Statistics

All statistical tests were performed with online calculators at www.vassarstats.net. The specific tests used for each experiment are mentioned in figure legends with results and p values.

## Supporting Information

S1 Fig Timelapse quantitation method used on each axon quantified for each timepoint. (a) Red SUM projection from the +UTR axon at 90 minutes after photoconversion shown in Fig 1 and S1 Movie, with the trace used to define pixels in the ratio SUM projection measured by ImageJ macro. (b) A chart of measurements generated by the ImageJ macro.
(TIF)

S2 Fig Injection with i3e4 MO gives retinotectal phenotype similar to e3i3 MO. (a) The i3e4 MO targeted to the i3e4 splice junction in Igf2bp1 pre-mRNA. (b-g) Transmitted light images of whole 3 dpf i3e4 MO-injected morphants. (h-u) 3D projections made from confocal z-stacks take with a 20x lens on a confocal microscope, of Tg(isl2b:mCherryCAAX)^zc23^ 3 dpf embryos stained with α-DsRed (red) and counterstained with TO-PRO-3 (blue), with one example each for uninjected (h), or injected with negative control MO (l), and three examples each injected with e3i3 MO (mild (i-k), moderate (p-r)) or i3e4 MO (mild (m-o), moderate (s-u)).
(TIF)

S3 Fig Cloning strategy for emGFP^A207K^ and expression constructs. (a) Cloning strategy used to introduce A207K point mutation into emGFP and (b) to generate pME-emGFP-linker. (c) Gateway entry clones used in LR reactions with pDEST-pA2, used to generate the cDNA expression constructs (d) used in the timelapse experiment (*isl2b*:Kaede-pA, *isl2b*:Kaede-β-actin3’UTR-pA) and in the dominant negative experiment (Fig 5, *isl2b*:emGFP-pA, *isl2b*:emGFP-linker-Igf2bp1wt-pA, *isl2b*:emGFP-linker-Igf2bp1Y399E-pA).
(TIF)

S4 Fig Quantification of the ratio of labeled RGC axons to labeled cell bodies in Fig 5. (a) Confocal projection (30x silicone immersion lens) of a 3 dpf embryo with transient expression of *isl2b*:emGFP, with labeled RGC axons (a red rectangle, b) and labeled cell bodies (yellow rectangle, c) that were counted. Axons were traced in the dorsal optic tract and tectum using the Fiji simple neurite tracer plugin (b). (c) The cell counter plugin in ImageJ was used to mark and count labeled cells in the contralateral retina. The ratio of labeled axons to labeled cell bodies per retinotectal projection was calculated (37.5%). Scale bar is 100 μm.
(TIF)

S1 Movie *In vivo* timelapse of green to red Kaede expression in RGC axons after photoconversion. One +UTR axon and one −UTR axon imaged in the optic tract *in vivo*, with a color map showing the ratio of green to red fluorescence. Frames are every 10 minutes, 0 to 90 minutes after photoconversion, for a total of 10 frames per axon.
(MP4)

S2 Movie Quantitation of green to red fluorescence ratio. Movie generated by the ImageJ macro used to measure the grayscale value of 150 pixels along the axon in the divide SUM projection defined by the retrograde trace made with Fiji (see methods), at 90 minutes post-photoconversion for the +UTR axon shown in S1 Movie and Fig 1. The circle follows the position of each pixel being measured and the histogram at the bottom represents the grayscale value in the ratio SUM projection, equal to the ratio of green to red fluorescence.
(MP4)

S3 Movie Editing used to isolate retina for counting AO-positive cells. 3D rendering, generated in FluoRender, of confocal z-stack (40x water immersion lens) of acridine orange staining, rotating around Y-axis, in a live 31 hpf uninjected embryo (similar to Fig 4I). The extraocular tissues and the lens (red) were erased with the paintbrush tool in ImageJ. The resulting stack (green) represented the retina alone. AO positive cells in each retina were counted in 3D using Imaris.
(MP4)

S1 Spreadsheet Data from Fig 1O and 1P. (XLSX)

S2 Spreadsheet Data from Fig 1Q–1S. (XLSX)

S3 Spreadsheet Graphs of data in Fig 1Q–1S. (XLS)

S4 Spreadsheet Data from Fig 4M. (XLSX)

S5 Spreadsheet Data from Fig 5D. (XLSX)

S1 Table Settings used for confocal imaging. (TIF)
