# Supplementary figures and images for: The RNA Binding Protein Igf2bp1 Is Required for Zebrafish RGC Axon Outgrowth In Vivo
Source: PLoS One. 2015 Sep 1;10(9):e0134751. doi: 10.1371/journal.pone.0134751 (PMC4556669; doi:10.1371/journal.pone.0134751)

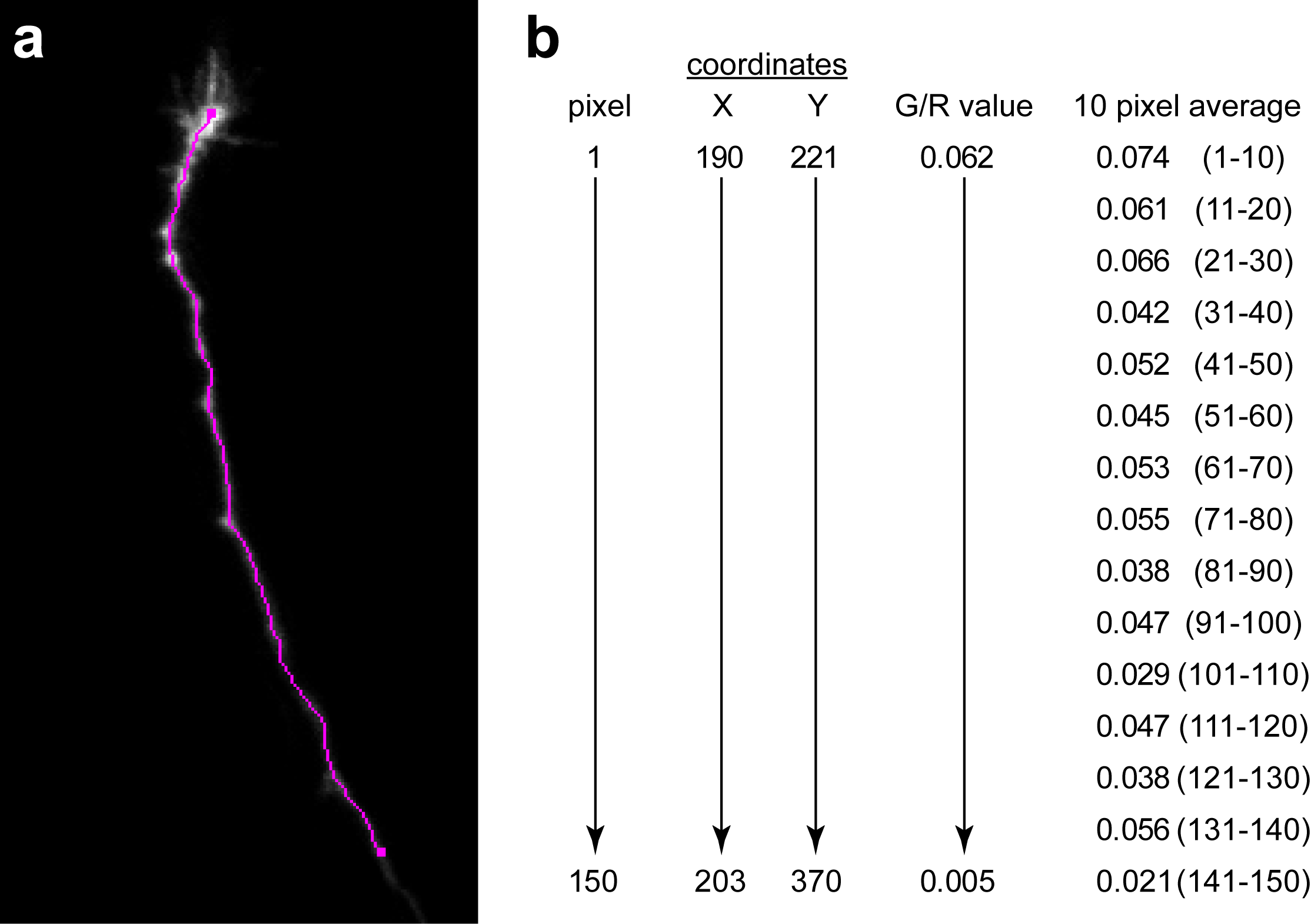

Supplement: S1 Fig — (a) Red SUM projection from the +UTR axon at 90 minutes after photoconversion shown in Fig 1 and S1 Movie, with the trace used to define pixels in the ratio SUM projection measured by ImageJ macro. (b) A chart of measurements generated by the ImageJ macro. (TIF) [file pone.0134751.s001.tif]

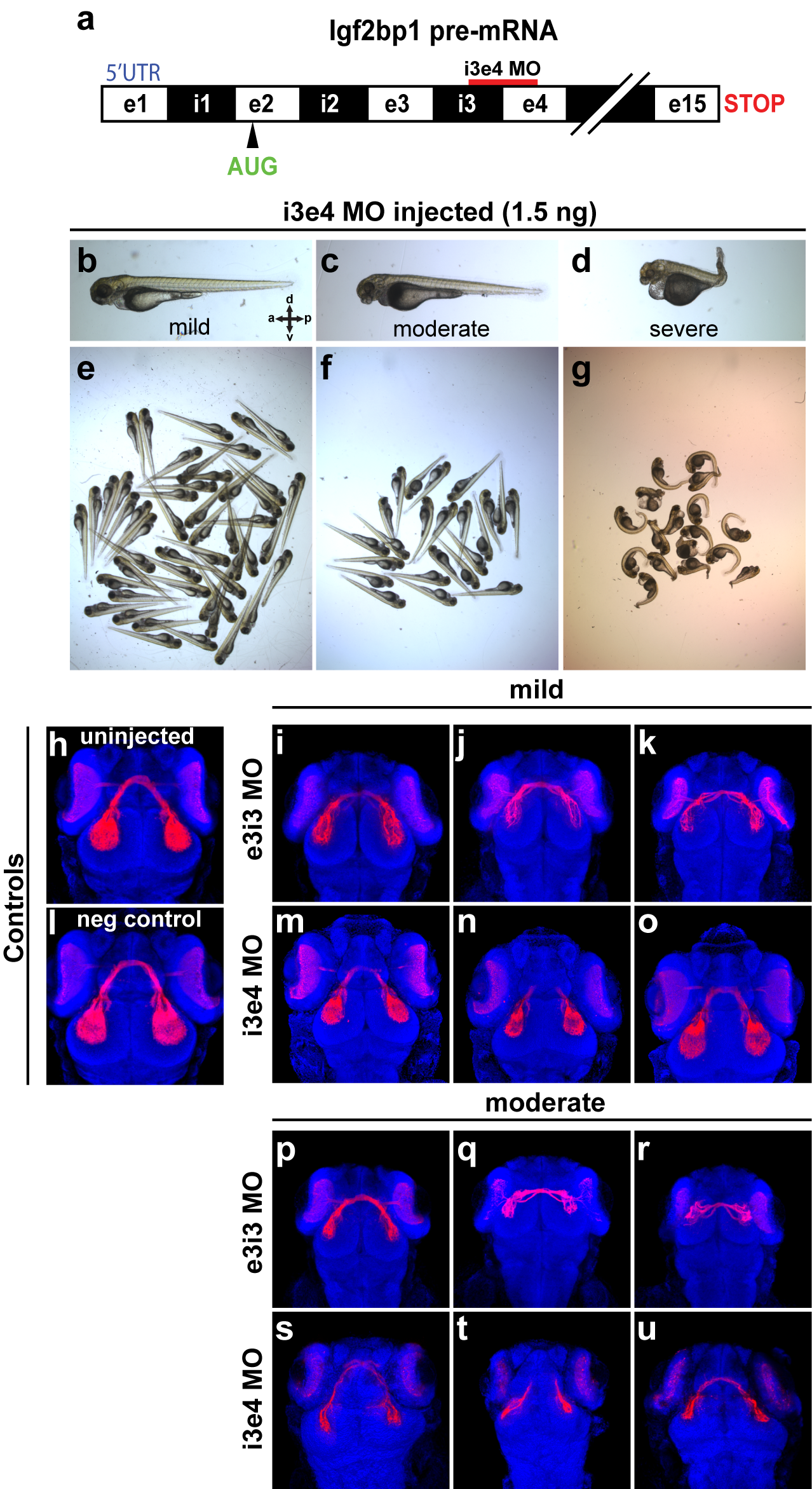

Supplement: S2 Fig — (a) The i3e4 MO targeted to the i3e4 splice junction in Igf2bp1 pre-mRNA. (b-g) Transmitted light images of whole 3 dpf i3e4 MO-injected morphants. (h-u) 3D projections made from confocal z-stacks take with a 20x lens on a confocal microscope, of Tg(isl2b:mCherryCAAX)zc23 3 dpf embryos stained with α-DsRed (red) and counterstained with TO-PRO-3 (blue), with one example each for uninjected (h), or injected with negative control MO (l), and three examples each injected with e3i3 MO (mild (i-k), moderate (p-r)) or i3e4 MO (mild (m-o), moderate (s-u)). (TIF) [file pone.0134751.s002.tif]

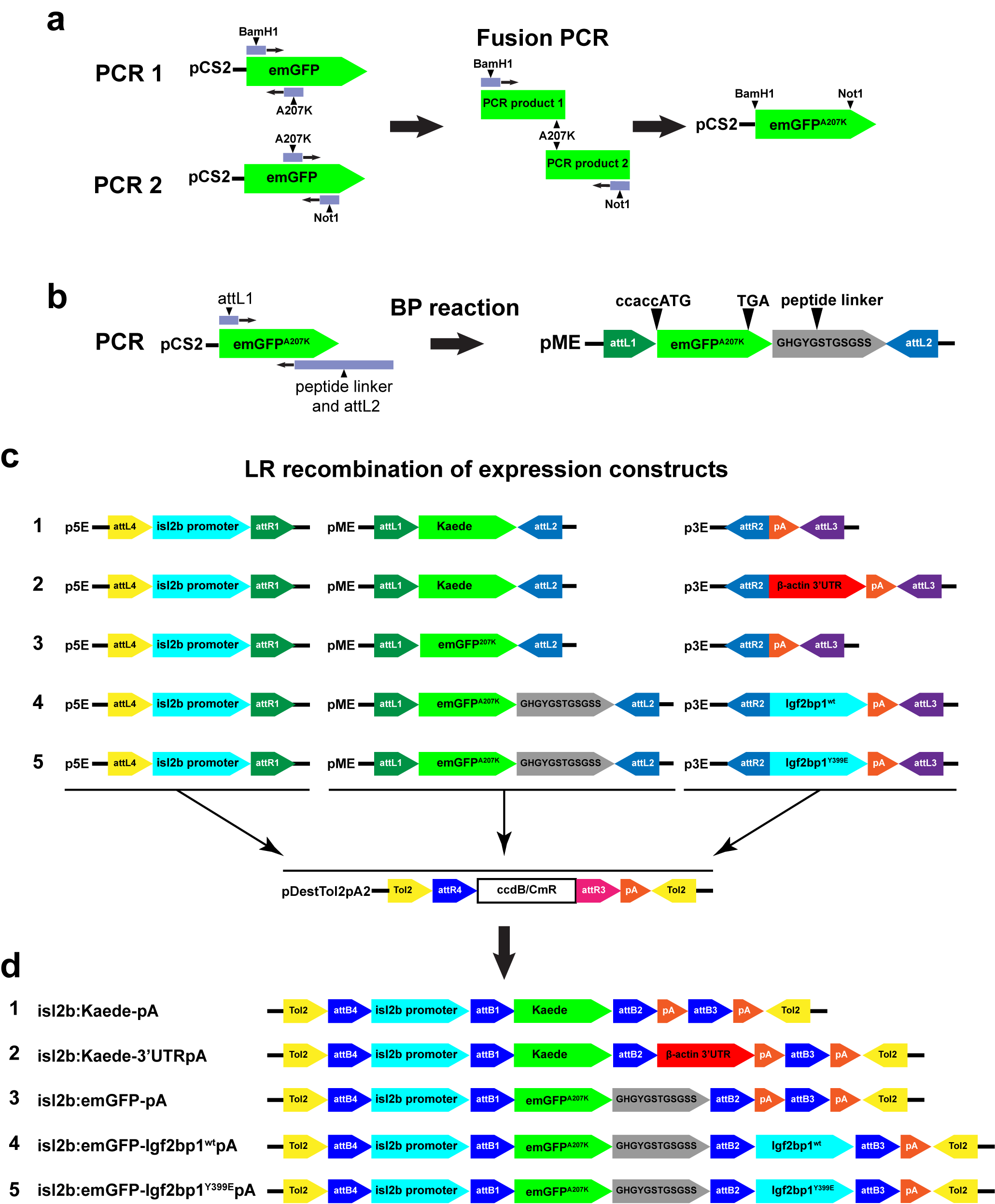

Supplement: S3 Fig — (a) Cloning strategy used to introduce A207K point mutation into emGFP and (b) to generate pME-emGFP-linker. (c) Gateway entry clones used in LR reactions with pDEST-pA2, used to generate the cDNA expression constructs (d) used in the timelapse experiment (isl2b:Kaede-pA, isl2b:Kaede-β-actin3’UTR-pA) and in the dominant negative experiment (Fig 5, isl2b:emGFP-pA, isl2b:emGFP-linker-Igf2bp1wt-pA, isl2b:emGFP-linker-Igf2bp1Y399E-pA). (TIF) [file pone.0134751.s003.tif]

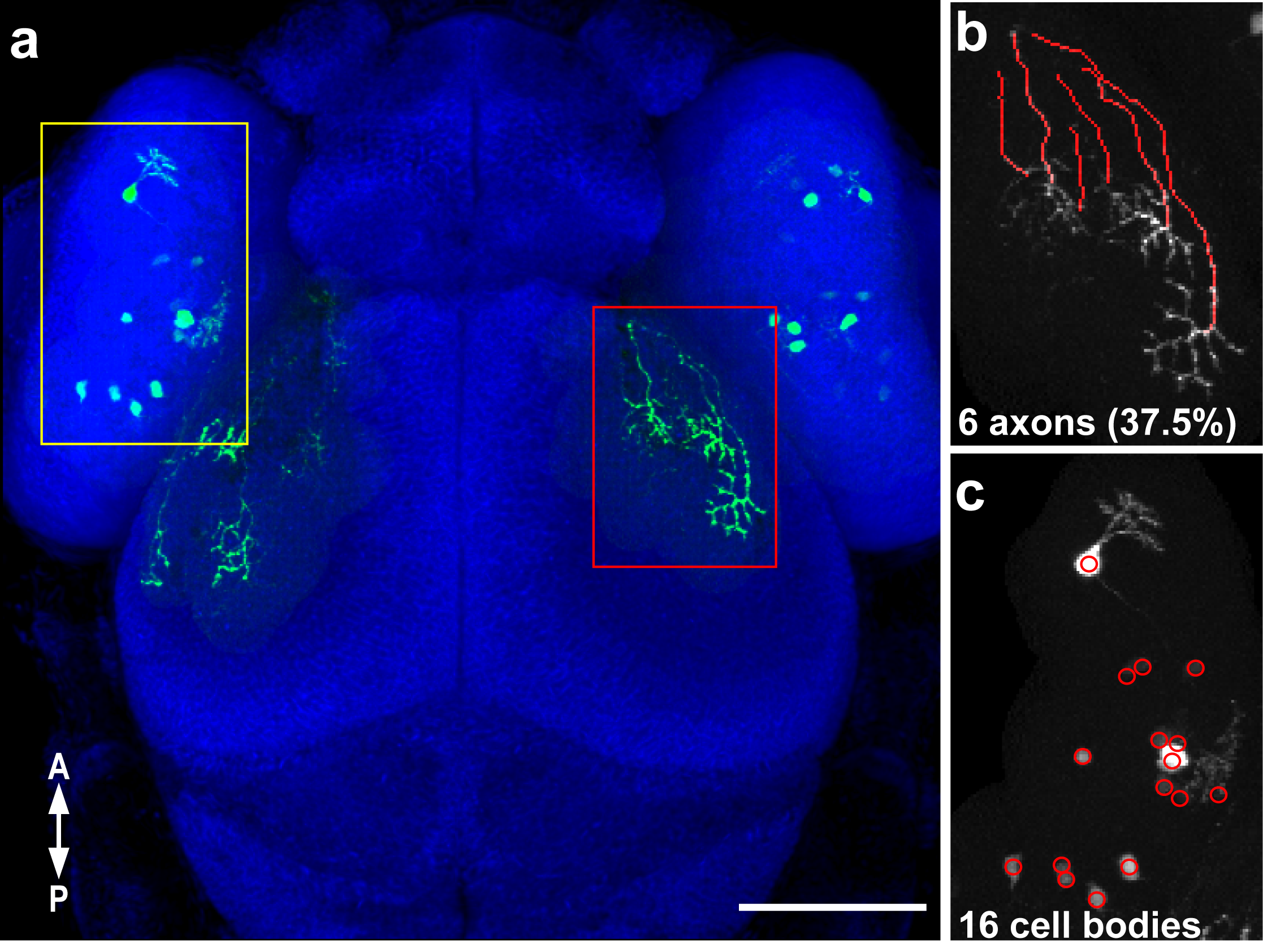

Supplement: S4 Fig — (a) Confocal projection (30x silicone immersion lens) of a 3 dpf embryo with transient expression of isl2b:emGFP, with labeled RGC axons (a red rectangle, b) and labeled cell bodies (yellow rectangle, c) that were counted. Axons were traced in the dorsal optic tract and tectum using the Fiji simple neurite tracer plugin (b). (c) The cell counter plugin in ImageJ was used to mark and count labeled cells in the contralateral retina. The ratio of labeled axons to labeled cell bodies per retinotectal projection was calculated (37.5%). Scale bar is 100 μm. (TIF) [file pone.0134751.s004.tif]

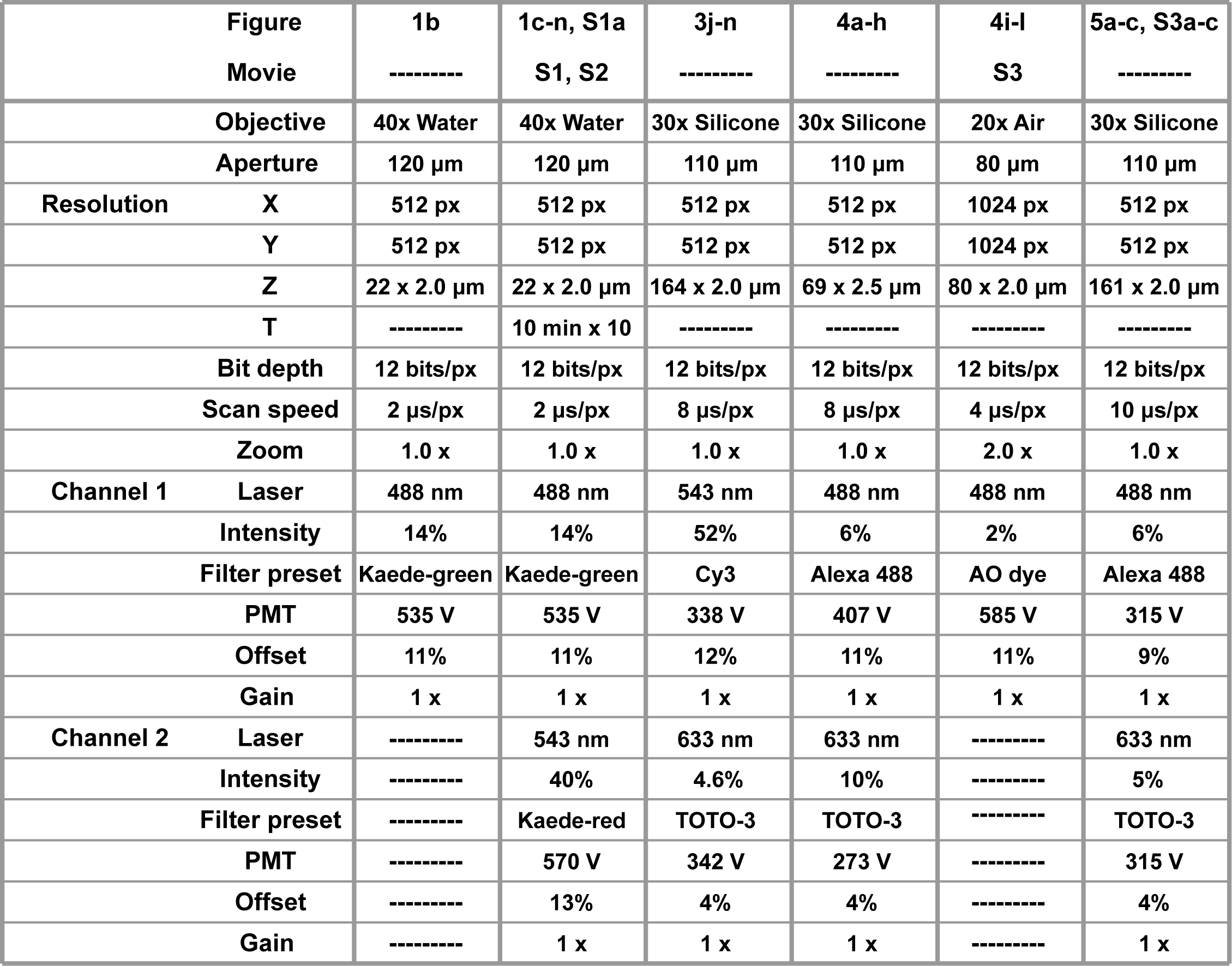

Supplement: S1 Table — (TIF) [file pone.0134751.s013.tif]
